# Study on Genotypes and Phenotypes of Neurodegenerative Diseases

**DOI:** 10.3390/genes15060786

**Published:** 2024-06-14

**Authors:** Claudia Ricci

**Affiliations:** Department of Medical, Surgical and Neurological Sciences, University of Siena, 53100 Siena, Italy; claudia.ricci@unisi.it

Neurodegenerative diseases are a heterogeneous group of age-related disorders that are characterised by the gradual degeneration or death of neurons in the central or peripheral nervous system. The prevalence of these diseases is increasing, partly due to an ageing population; as a result, the economic burden on healthcare systems is also growing. Recent advances in neurobiology and neurogenetics have provided valuable insights into the pathogenesis of neurodegenerative diseases.

Genetic factors play a key role in the pathogenesis of a number of neurodegenerative diseases, acting both as monogenic causes in the inherited forms and as modulating factors in the multifactorial/sporadic forms. The latest advances in cost-effective genetic analysis have significantly increased our knowledge of the genetic basis of several neurodegenerative diseases; in particular, omics studies have provided a better understanding of the mechanisms underlying their pathogenesis.

A common feature of neurodegenerative diseases is that they exhibit a degree of genetic heterogeneity; in other words, the presentation and severity of the disease can vary from person to person. Sometimes, different variants in different genes can even cause the same phenotype. On the other hand, the same mutation can be associated with phenotypic heterogeneity, even within the same family. In some cases, however, a specific variant may be associated with a common phenotype, which may be useful for diagnostic and prognostic purposes. In the era of precision medicine, a better characterisation of genotype–phenotype correlations may help to improve therapeutic approaches, assess individual drug responses, and guide gene-targeted clinical trials.

Several papers in this Special Issue are based on the use of omics technologies to study the basis of neurodegenerative diseases.

In their study, Katarzyna Gaweda-Walerych and colleagues analysed the phenotypic–genetic correlations in a patient diagnosed with early onset corticobasal syndrome with progressive non-fluent aphasia (CBS-PNFA) using a whole-exome sequencing (WES) approach [1]. They identified rare single heterozygous variants in the *ATP7B*, *SORL1*, *SETX*, and *FOXP1* genes, extending the complex clinical spectrum associated with variants in known disease genes and also supporting the hypothesis of oligogenic inheritance. In addition, the study highlights the possible relevance of the *FOXP1* gene in relation to progressive language apraxia in adulthood, whereas previously this gene has only been associated with neurodevelopmental language disorders.

In some cases, the use of large-scale genome-wide association studies (GWASs) has produced inconclusive results. In Alzheimer’s disease (AD), for example, the problem of “missing heritability” is still a major challenge. Dandan Chen and colleagues have addressed this issue by focusing on epistasis [2], whereby the effect of a mutation depends on the genetic background in which it occurs. Their study focused on identifying epistasis between two-marker interactions with marginal main effects across the genome, employing cerebrospinal fluid (CSF) P-tau as a quantitative trait. Using this approach, they identified 758 statistically significant SNP pairs and several highly significant SNP–SNP interactions that explained a relatively high amount of variance at the P-tau level. In addition, 331 AD-related genes were identified, and 10 gene–gene interaction pairs were replicated in the PPI network, showing associations with AD in terms of neuroinflammation, neurodegeneration, neuronal cell activation, and brain development, leading to a cognitive decline in people with AD. Overall, this study may, therefore, open up new perspectives to complement traditional GWASs and to help explain some of the “missing heritability” of Alzheimer’s disease.

AD was also studied in the paper by Yury Loika and colleagues [3]. In this case, the authors aimed to identify clusters of cardiovascular traits that share genetic factors with Alzheimer’s disease. They performed a univariate exome-wide association study and a pair-wise pleiotropic analysis focusing on AD and 16 cardiovascular traits (6 diseases and 10 cardio-metabolic risk factors) using 188,260 samples from the UK Biobank (UKB). The analysis identified nine genetic markers in the *APOE* gene region, and four loci mapped to the *CDK11*, *OBP2B*, *TPM1*, and *SMARCA4* genes that showed associations with AD and pleiotropic associations. Using hierarchical cluster analysis, the authors grouped the phenotypes from these pleiotropic associations into seven clusters. Interestingly, all the AD protective alleles were associated with an increased weight and a risk of diabetes mellitus. The overall results of the study suggest that common genetic factors between Alzheimer’s disease and cardiovascular traits are influenced in an antagonistic way.

Furthermore, Sangeetha Vishweswaraiah and colleagues investigated the interaction between the metabolome and the epigenome in Huntington’s disease (HD) [4]. They performed metabolomic profiling of human post-mortem brain tissue (striatum and frontal lobe) and DNA methylome profiling using the same frontal lobe tissue. Among several perturbed metabolites and differentially methylated loci, they identified aminoacyl-tRNA biosynthesis as being the most significantly perturbed pathway, correlating with two CpGs of the *SEPSECS* gene. This study reveals important links between molecular biomarkers and adds to the knowledge of the metabolic changes that drive the progression of HD.

An important aspect of neurodegenerative diseases is the possible existence of genotype–phenotype correlations. In their article, Lea Hentrich and colleagues describe two patients with progressive myoclonus epilepsy with neurodegeneration that is associated with biallelic variants of the Golgi SNAP receptor complex member 2 gene (*GOSR2*) [5]. The authors report the clinical findings and a genetic characterisation of both patients. The study extends the genotype–phenotype spectrum of *GOSR2*-related disorders and suggests that the *GOSR2* gene should be included in the consideration of the monogenetic causes of dystonia, global developmental delay, and seizures.

The availability of affordable disease models is fundamental to the ongoing search for novel therapeutic approaches. Camille Bouchard and colleagues reported the discovery of a suitable mouse model for Friedreich’s Ataxia (FRDA), which is a progressive neurodegenerative disease caused by a triplet repeat in the frataxin gene (*FXN*) that leads to a reduced expression of the frataxin protein [6]. The previously used mouse model (YG8sR mice) showed a milder phenotype compared to human patients, limiting the ability to study the impact of therapy on the phenotype. The authors compared two different mouse models—a modified YG8sR model injected with an AAV encoding an shRNA that targets the human frataxin gene, and the YG8-800, a new mouse model with a human transgene containing 800 GAA repeats. Behavioural tests showed that the mild phenotype of YG8sR mice can be enhanced by injecting them with an AAV expressing an shRNA targeting frataxin, and that the phenotype of YG8-800 mice is comparable to the human ataxic phenotype.

The role of genetic factors in contributing to ethnic differences in health and disease is becoming increasingly recognised. Anusha Mamidipaka and colleagues examined the demographic, optic disc, and genetic risk factors for a large cup-to-disc ratio (LCDR) presentation and its subsequent progression to visual impairment or blindness in a large cohort of individuals of African ancestry that were recruited from the Primary Open-Angle African American Glaucoma Genetics (POAAGG) study [7]. No significant associations were found between genetic variants and LCDR or blindness, while previous glaucoma surgery, increased intraocular pressure, decreased mean deviation, and decreased pattern standard deviation were statistically significant risk factors for blindness in LCDR eyes. These findings highlight the importance of the close monitoring of intraocular pressure and visual function in POAG patients of African descent, especially those with LCDR, in order to preserve visual function.

The importance of risk factors for disease development is also emphasised in one of the four reviews in the Special Issue. Edward O. Olufunmilayo and colleagues [8] review the role of the Src homology 2 (SH2) domain-containing inositol 50 phosphatase 1 (SHIP1) protein, encoded by the *INPP5D* (inositol polyphosphate-5-phosphatase D) gene, in Alzheimer’s disease. *INPP5D* expression and SHIP1 activity have been shown to be important risk factors for the development of late-onset Alzheimer’s disease, as the various mechanistic actions of SHIP1 in microglia result in the attenuation of signalling and, as a consequence, some of the beneficial functions of these cells. In addition, polymorphisms in the *INPP5D* gene have been found to be associated with a significantly increased risk of AD; *INPP5D*/SHIP1 may, therefore, be an interesting therapeutic target for the disease.

Another review focuses on the role of IgLON cell adhesion molecules in neurodegenerative diseases. Salluzzo and colleagues [9] describe the structure, functional domains, interactions, and activities of the IgLON family, a class of adhesion molecules consisting of five members; they also analyse the available data and suggest a role for IgLONs in neuropsychiatric disorders. The authors highlight the evidence that is already available and the mechanisms that require further in-depth investigation.

Martin N. Ivanov and colleagues discuss the current understanding of the involvement of the apelinergic system in the brain physiology in health and disease [10]. The authors report on the structure and function of the apelin receptor (APLNR) and its ligands—apelin and ELABELA. They also describe the role of the apelinergic system in a typical brain, as well as in several pathological conditions including Parkinson’s and Alzheimer’s diseases, in which apelin peptides appear to exert protective effects.

Another important issue for neurodegenerative disease research is the identification of outcome measures and biomarkers for clinical trials [11]. Sue-Faye Siow and colleagues conducted a scoping review of outcome measures and biomarkers in hereditary spastic paraplegia (HSP) with the aim of providing recommendations for future studies. They discuss the advantages and limitations of commonly used outcome measures and suggest areas for further research. Overall, the authors report a significant variability and inconsistency in using outcome measures with limited longitudinal data. Given the emergence of several candidate HSP therapies in recent years, they suggest that there is an urgent need to further develop a core set of validated and standardised outcome measures for use in HSP clinical trials to test the efficacy of these therapies.

In conclusion, this Special Issue provides an overview of research into the genotypes and phenotypes of neurodegenerative diseases, ranging from the use of cutting-edge omics techniques to the identification of biomarkers and the characterisation of new experimental models. The picture that emerges is one of ongoing research efforts to more effectively combat these devastating diseases and to identify potential future therapeutic targets.
